# Gut microbiota regulates optic nerve fiber myelination

**DOI:** 10.3389/fcell.2025.1526855

**Published:** 2025-02-20

**Authors:** Giulia Ronchi, Davide Pellegrino, Marwa El Soury, Olga Amato, Francesco Gaia, Sajjad Farzin, Raffaele Nuzzi, Marijana Basic, Silvia Bolsega, Stefano Geuna, Matilde Cescon, Kirsten Haastert-Talini, Giovanna Gambarotta

**Affiliations:** ^1^ Department of Clinical and Biological Sciences & Neuroscience Institute Cavalieri Ottolenghi (NICO), University of Torino, Torino, Italy; ^2^ Department of Neurosciences “Rita Levi Montalcini”, Eye Clinic, University of Torino, Torino, Italy; ^3^ Institute for Laboratory Animal Science and Central Animal Facility, Hannover Medical School, Hannover, Lower-Saxony, Germany; ^4^ Department of Molecular Medicine, University of Padova, Padova, Italy; ^5^ Institute of Neuroanatomy and Cell Biology, Hannover Medical School, Hannover, Lower-Saxony, Germany; ^6^ Centre for Systems Neuroscience (ZSN), Hannover, Lower-Saxony, Germany

**Keywords:** gnotobiotic mice, microbiota, myelin, oligodendrocytes, germ-free mice

## Abstract

**Introduction:**

Recent evidence supports the hypothesis of an association between gut microbiota and the pathogenesis of retinal and eye diseases, suggesting the existence of a gut-eye axis. However, no data are available on the possible effect of the gut microbiota on the optic nerve fiber maturation and myelin development.

**Methods:**

We investigated the impact of gut microbiota on the optic nerves collected from neonatal and young adult germ-free (GF), gnotobiotic (stably colonized with 12 bacteria strains, OMM12) and control (colonized with a complex gut microbiota, CGM) mice, by performing stereological and morphoquantitative analyses with transmission electron microscopy and gene expression analysis by quantitative real-time PCR.

**Results:**

Young adult GF and OMM12 optic nerve axons are smaller and hypermyelinated compared to CGM ones, while no such differences were detected in neonatal optic nerves. The transcription factors *Olig1*, *Olig2*, and *Sox10* (oligodendrocyte myelination positive regulators) are downregulated in CGM and OMM12 young adult mice compared to the respective neonates. Such developmental downregulation was not observed in GF optic nerves, suggesting that the absence of the gut microbiota prolongs the stimulation of optic nerve fiber myelination, possibly through mechanisms that are yet to be identified.

**Discussion:**

Altogether, these data underscore the gut microbiota pivotal role in driving optic nerve myelination, contributing to our knowledge about both the gut-eye axis and the gut-brain axis, and opening new horizons for further investigations that will explore the role of the microbiota also in pathologies, injuries and regeneration associated with the optic nerve.

## Introduction

The optic nerve is the second cranial nerve responsible for transmitting special sensory information for vision from the retina to the brain, and contains only afferent (sensory) fibers. These fibers are the extensions of ganglion cells located in the ganglion cell layer of the retina (Smith and Czyz, 2024). The optic nerve also contains different types of glial cells, namely, oligodendrocytes, astrocytes, microglia, and neuron glial antigen 2 (NG2) expressing cells, which support the functionality of the optic nerve fibers (Yazdankhah et al., 2021). Damage to the optic nerve or along the visual pathway can cause a variety of visual field defects (Sarkies, 2004; Atkins et al., 2008).

The optic nerve is considered a central nervous system (CNS) structure, like the cranial nerves III-XII. The optic nerve is derived from the outpouching optic vesicles from embryonic diencephalon during development. As a CNS structure, the optic nerve is myelinated by oligodendrocytes and is ensheathed in the three meningeal layers (dura mater, arachnoid mater, and pia mater) (Smith and Czyz, 2024).

Myelination of axons by specific glial cells is a dynamic process spanning from early life to adulthood in both the peripheral nervous system (PNS) and CNS, and it is crucial for regulating motor, sensory and higher-order cognitive functions (Almeida and Lyons, 2017; Cristobal and Lee, 2022). Over the past decade, there has been a significant surge in research on the gut-microbiota-brain axis, revealing compelling connections between the gut microbiota composition and the structure and function of the brain. Recent findings underscored the pivotal role of the gut microbiota in regulating CNS myelination (Keogh et al., 2021; Lynch et al., 2021; Needham et al., 2022; Sharvin et al., 2023), while we could demonstrate a direct connection between gut microbiota composition and myelination in the PNS (Calabrò et al., 2023; Cescon et al., 2024).

The gut microbiota consists of the collection of bacteria, archaea, viruses and eukarya inhabiting the gastrointestinal tract and its composition undergoes dynamic changes from birth to adulthood (Afzaal et al., 2022). Various factors, including the mode of colonization, antibiotic use, dietary habits, lifestyle, and age, contribute to the continuous modifications in the gut microbiota composition throughout an individual’s life (Thursby and Juge, 2017). It is also noteworthy that with regard to the developing nervous system, the maternal gut microbiome plays a crucial role (Vuong et al., 2020; Lu et al., 2020). The gut microbiota - more specifically, its “products” and metabolites - plays a pivotal role in sustaining host health, and alterations in its composition have been linked to a spectrum of diseases, including inflammatory bowel diseases, autoimmune disorders, neurological conditions, cardiovascular and liver diseases, and more (Cryan et al., 2019; Masenga et al., 2022; Miyauchi et al., 2023). Investigating the impact of the gut microbiota and its metabolites on host health represents therefore one of the most active fields of biomedical research and can be expected to enhance our understanding of disease mechanisms and to inspire the development of innovative therapeutic strategies in disease management.

The relationship between the eye and the gut microbiota is an emerging area of research (Fu et al., 2023) and recent studies have highlighted a potential association between gut microbiota and ocular health, suggesting the existence of a “gut–eye axis” implicated in the development and progression of multiple ocular conditions including uveitis, age-related macular degeneration, diabetic retinopathy, dry eye, glaucoma and chalazion (Napolitano et al., 2021; Scuderi et al., 2021; Campagnoli et al., 2023; Nguyen et al., 2024). Together with the gut microbiota, also the ocular surface microbiota has been shown to influence the occurrence of various eye diseases, such as blepharitis, conjunctivitis, keratitis, trachoma and dry eye syndrome (Song et al., 2024; Xue et al., 2021). Despite the increasing number of studies on this topic, to date the literature lacks data regarding the influence of gut (or others) microbiota on the maturation of optic nerve fibers and the development of their myelination. To fill this gap, in the current study, we analyzed optic nerves from neonatal and young adult germ-free (GF), gnotobiotic (stably colonized with 12 bacteria strains, Oligo-Mouse-Microbiota 12, OMM12), and mice characterized by a specific pathogen-free, but complex, gut microbiota composition (CGM).

## Materials and methods

### Mice and sample collection

Samples were harvested from the same animals used for a recently published previous study (Cescon et al., 2024). Briefly, neonatal (P8-14) and young adult (63–67 days old) germ-free (GF), gnotobiotic (colonized with Oligo-Mouse-Microbiota 12, OMM12 (Eberl et al., 2019)], and specific pathogen-free C57BL6/JZtm (harboring specific-pathogen free, complex gut microbiota, CGM) mice were obtained from the Central Animal Facility at Hannover Medical School, Hannover, Germany. By colonising GF C57BL6/JZtm mice with their respective microbiota, microbiota-colonised mice (CGM and OMM12) were generated. The colonization procedure was repeated every 10 generations to avoid the formation of substrains. The breeding and maintenance of GF and OMM12 mice was performed in plastic film isolators (Metall + Plastik GmbH, Radolfzell-Stahringen, Germany) located in a room with a controlled environment (20°C–22°C, 50%–55% humidity) and 12-h light/dark cycles. The animals were collected sequentially over a period of several months from stable breeding colonies (CGM, OMM12 and GF) depending on their availability. The animals were sacrificed by decapitation (young adult ones after inducing anesthesia in CO_2_ atmosphere). Further details about gut microbiota colonization procedure, breeding and maintenance, as well as protocols for screening of GF and OMM12 colonies and results of the shallow shotgun metagenomics we carried out on young adult CGM and OMM12 mice fecal pellets are available in our previous published work (Cescon et al., 2024).

All procedures were performed in accordance with the German Animal Welfare Legislation and the principles of the Basel Declaration and recommendations of Directive 2010/63/EU, and were approved by the local Institutional Animal Care and Research Advisory Committee and registered with the Animal Care Committee of Lower-Saxony, Germany. Breeding and animal husbandry was registered under the number 42500/1H according to §11 of the German protection of animals act (TierSchG). Animal sacrifice for scientific purposes was announced to the authorities under the numbers §4 2021-289 (postnatal mice) and §4 2017-171 (young adult mice).

### Stereological and morphometric analysis of optic nerve fibers

A total of 30 fresh optic nerves collected from 15 neonates and 15 young adult mice were analyzed, with n = 5 optic nerves derived from n = 5 different animals for each experimental group/age (CGM, OMM12, GF).

Nerve samples were fixed using Karnovsky solution (2% paraformaldehyde, 2.5% glutaraldehyde in 0.2 M sodium cacodylate buffer, pH 7.3, 24 h), washed (0.1 M sodium cacodylate, 7.5% sucrose), post–fixed (1% osmium tetroxide for 1.5 h), and then embedded in epon as previously described (Haastert-Talini et al., 2013).

2.5 μm thick semi-thin transverse sections were cut using an Ultracut UCT ultramicrotome (Leica Microsystems, Germany); sections were stained with 1% Toluidine blue and blindly analyzed with a DM4000B microscope equipped with a DFC320 digital camera and an IM50 image manager system (Leica Microsystems, Germany) to measure the nerve cross-sectional area (×20 magnification).

Stereological and morpho-quantitative analyses (total fiber number, fiber density and size parameters - fiber and axon diameter, myelin thickness and *g*-ratio) were performed on ultrathin sections (70-nm-thick) using a JEM-1010 transmission electron microscope (JEOL, Tokyo, Japan) equipped with a Mega-View-III digital camera and a Soft-Imaging-System (SIS, Münster, Germany). Images were acquired at a magnification of ×12,000 and then analyzed using the ImageJ software.

### Quantitative real-time PCR (qRT-PCR) analysis

RNA from snap frozen optic nerve samples (from the opposite eye as above) was isolated using the TRIzol Reagent (Life Technologies) following manufacturer’s instructions. RNA extracted from each sample was quantified using NanoDrop 1000 Spectrophotometer (Thermo Scientific) and its purity was evaluated by reading the absorbance at 260, 280 and 230 nm.

Complementary DNA (cDNA) of each sample was obtained by reverse-transcription (RT). To reverse transcribe RNA to cDNA, 0.4 μg RNA for each sample were reverse-transcribed in a 25 μL total reaction volume containing 1 mM dNTPs, 1x RT-buffer, 0.10 μg/μL bovine serum albumin (BSA), 0.05% Triton, 7.5 μM Random Hexamer Primers, 200 U reverse transcriptase (RevertAid Thermo Scientific Fermentas, #EP0441), 40 U RNase inhibitor (Ribolock Thermo Scientific Fermentas #E00381). To perform the reverse transcriptase reaction, Thermocycler was set at 25°C for 10 min, 42°C for 90 min, 70°C for 10 min. The obtained cDNA was diluted 1:10 in a final volume of 250 μL nuclease free water and finally stored at −20°C.

To quantify cDNA and determine gene expression, quantitative real-time Polymerase Chain Reaction (qRT-PCR) was performed with an ABI prism 7300 (Applied Biosystems) detection system using Sybr Green chemistry. Reactions were run in duplicates for each sample on 96-well optical PCR plates (Bio-Rad). In each well a qRT-PCR was carried out in a reaction volume of 20 μL containing 5 μL of diluted cDNA, 1x iTaq Universal SYBR Green Supermix (Bio-Rad) and 300 nM forward and reverse primers (Life Technology). All primers were designed using ANNHYB software (http://www.bioinformatics.org/annhyb/) and synthesized by Invitrogen (Life Technologies Europe BV, Monza, Italy). Primer sequences are reported in Table 1. After an initial denaturation step for 30 s at 95°C, denaturation in the subsequent 40 cycles was performed for 15 s at 95°C followed by primer annealing and elongation at 60°C for 1 min. The dissociation curves obtained were routinely analyzed to check the quality of the reaction.

**TABLE 1:** List of selected primers used in the qRT-PCR reaction, showing the NCBI accession number and the length of the amplification product obtained from the reaction.

Gene	NCBI accession number	Primers	Sequence 5′-3′	Amplification length
*Tbp*	NM_013684.3	mrTbp FW	GAT​CAA​ACC​CAG​AAT​TGT​TCT​CC	106 bp
		mrTbp REV	GGG​GTA​GAT​GTT​TTC​AAA​TGC​TTC	
*Olig1*	NM_016968.4	mrOlig1 FW	GTGTGAACGCGGCTCCCG	100 bp
		mrOlig1 REV	GGT​GGC​TGC​CTG​TAA​CCC​AC	
*Olig2*	NM_016967.2	mrOlig2 FW	GACTCGGACGCCAGCCTG	107 bp
		mrOlig2 REV	CCT​CCT​GTG​AAG​CCG​CTG​C	
*Sox2*	NM_011443.4	mrSox2 FW	CTC​GGA​GAT​CAG​CAA​GCG​CC	111 bp
		mrSox2 REV	TGC​TCC​TTC​ATG​TGC​AGA​GCG	
*Sox10*	NM_011437.1	mrSox10 FW	CCA​TGT​CAG​ATG​GGA​ACC​CAG​AGC	80 bp
		mrSox10 REV	CTC​TGT​CTT​TGG​GGT​GGT​TGG​AGG	
*Myrf*	NM_001033481	mMyrf FW	CCC​TAT​GCC​CCA​GGC​ACA​C	110 bp
		mMyrf REV	GTC​TCC​GGG​GTT​ATG​GTG​CG	
*Pten*	NM_008960.2	mPten FW	GGC​GGA​ACT​TGC​AAT​CCT​CAG​TTT​G	120 bp
		mPten REV	CAA​TGG​CTG​AGG​GAA​CTC​AAA​GTA​CAT​G	
*c-Jun*	NM_021835.3	mrJun FW	ACG​ACC​TTC​TAC​GAC​GAT​GCC​C	116 bp
		mrJun REV	GGG​TCG​GCC​AGG​TTC​AAG​G	
*Mbp*	NM_001025251.2	mrMbp FW	GGA​CCC​AAG​ATG​AAA​ACC​CAG​TAG​TCC	81 bp
		mMbp REV	CCT​TCC​CTT​GGG​ATG​GAG​GTG​G	
*Plp1*	NM_011123.4	mPlp FW	GAG​CGG​GTG​TGT​CAT​TGT​TTG​GG	119 bp
		mPlp1_REV	GTA​CAC​AGG​TAC​AGC​CGA​GCA​G	
*P2Y12R*	NM_001357008.1	mP2Y12R FW	GCC​AGT​CTG​CAA​GTT​CCA​CTA​ACT​AG	118 bp
		mP2Y12R REV	GAA​GGT​GGT​ATT​GGC​TGA​GGT​GG	
*Iba1*	AK006562.1	mIba1 FW	GGG​GAT​CAA​CAA​GCA​ATT​CCT​CGA​TG	160 bp
		mIba1 REV	CCC​AAG​TTT​CTC​CAG​CAT​TCG​CTT​C	
*CD68*	BC021637.1	mCD68 FW	ACT​TCG​GGC​CAT​GTT​TCT​CT	138 bp
		mCD68 REV	GCT​GGT​AGG​TTG​ATT​GTC​GT	

m, indicates primer compatible for mouse; r, indicates primer compatible for rat; mr, indicates primer compatible for both species.

Data obtained with qRT-PCR experiments were quantified using the 2^−ΔΔCt^ relative quantification method (Livak and Schmittgen, 2001).

For data normalization, the threshold cycle number (Ct) values obtained of both the calibrator and the samples of interest were normalized to the housekeeping gene *Tbp* (TATA-box binding protein).

### Protein extraction and western blot analysis

Optic nerve total proteins were extracted using TRIzol reagent (Life Technology) after RNA extraction according to the manufacturer’s instructions. Then, the protein pellet was dissolved in a boiling LaemLi buffer (2.5% sodium dodecyl sulphate, 0.125 M Tris-HCl, pH 6.8). The Bicinchoninic Acid assay kit (Sigma-Aldrich, Merck) was used to determine protein concentration and equal amounts of proteins (10 µg) were resolved by 7.5% precasted SDS-PAGE (Bio-Rad) and blotted on supported nitrocellulose membrane (#1620094, Bio-Rad). Western blot analysis was carried out as previously described (Gambarotta et al., 2004). Primary antibodies used were: rabbit anti-AKT (1:1000, Cell Signaling, #9272); mouse anti-GAPDH (1:20000; Applied Biosystem, Ambion, #AM300); secondary antibodies used were HRP-linked anti-rabbit (Cell Signaling Technology, #7074) and anti-mouse (Cell Signaling Technology, #7076), both diluted 1:15000 in 5% non-fat dry milk in TBS-T. Bands were detected using ECL substrate (#170-5061, Bio-Rad), collected with Chemidoc, quantified with Image Lab Software (Bio-Rad, California, United States). The AKT band intensity was normalized to GAPDH.

### Statistical analysis

Statistical analyses were performed using GraphPad Prism Version 8 (GraphPad Software, San Diego, CA, United States). Normal distribution was tested using the Shapiro-Wilk test. For parametric data, the statistical significance was determined by one-way ANOVA followed by Tukey’s multiple comparisons *post hoc* test. Non-parametric data were subjected to the Kruskal–Wallis test followed by Dunn’s multiple comparisons *post hoc* test or Mann-Whitney test, as indicated. P values were considered significant at a p value ≤0.05. Data are expressed as the mean ± SEM.

## Results

We examined the optic nerves of neonatal and young adult GF, OMM12 and CGM mice to determine whether there were alterations in nerve fiber morphology, number, dimension and myelin sheath morphology and thickness in relation to the different gut microbiota composition.

**FIGURE 1:**
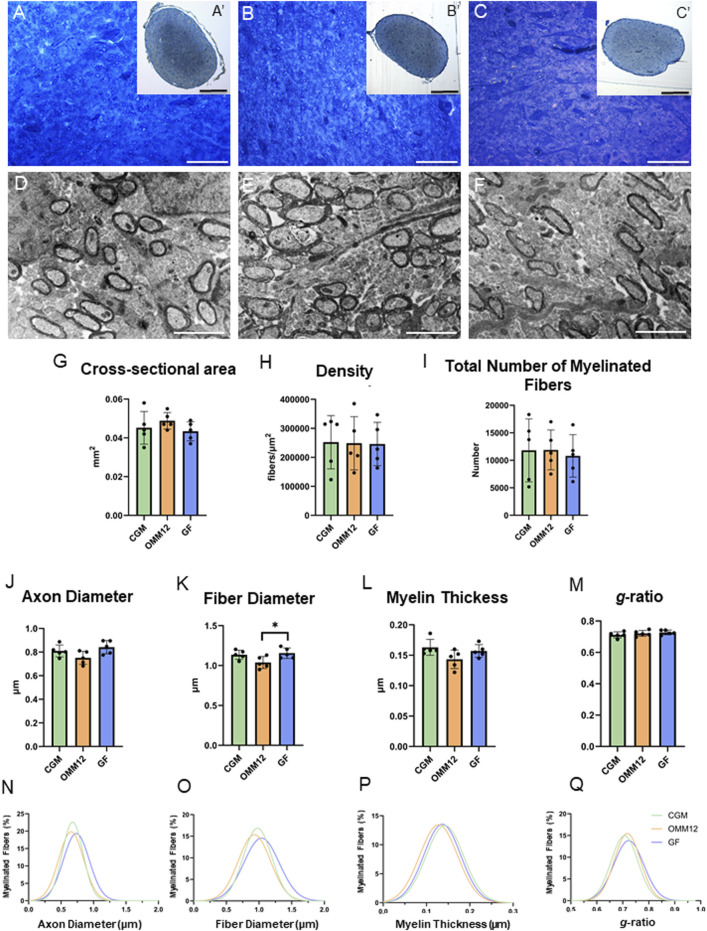
Neonatal optic nerves showed no morphological differences among groups. **(A–C; A′-C′)**. Toluidine blue-stained cross sections of optic nerves obtained from CGM **(A, A′)**, OMM12 **(B, B′)** and GF **(C, C′)** mice, at lower **(A′, B′, C′)** and higher **(A–C)** magnification. **(D–F)** Electron-microscopy images of optic nerves from CGM **(D)**, OMM12 **(E)**, GF **(F)**. A′, B′, C′ Scale Bar = 100µm; **(A, B, C)** Scale bar = 20 μm; **(D, E, F)** Scale bar = 2 μm; **(G–M)** Stereological and morpho-quantitative results: cross-sectional area **(G)**, nerve fiber density **(H)**, total number of myelinated fibers **(I)**, axon diameter **(J)**, fiber diameter **(K)**, myelin thickness **(L)** and *g*-ratio **(M)**. Parametric data were subjected to One-Way ANOVA followed by Tukey’s multiple comparisons *post hoc* test; bar graphs depict the mean ± SEM; *p ≤ 0.05 (n = 5 animals per group). **(N–Q)** Regression curves (Gaussian equation, not given) of percentile distribution for axon diameter **(N)**, fiber diameter **(O)**, myelin thickness **(P)** and *g*-ratio **(Q)**. For each experimental group, the total number of analysed optic nerve fibers were: CGM n = 536; OMM12 n = 579; GF n = 631.

**FIGURE 2:**
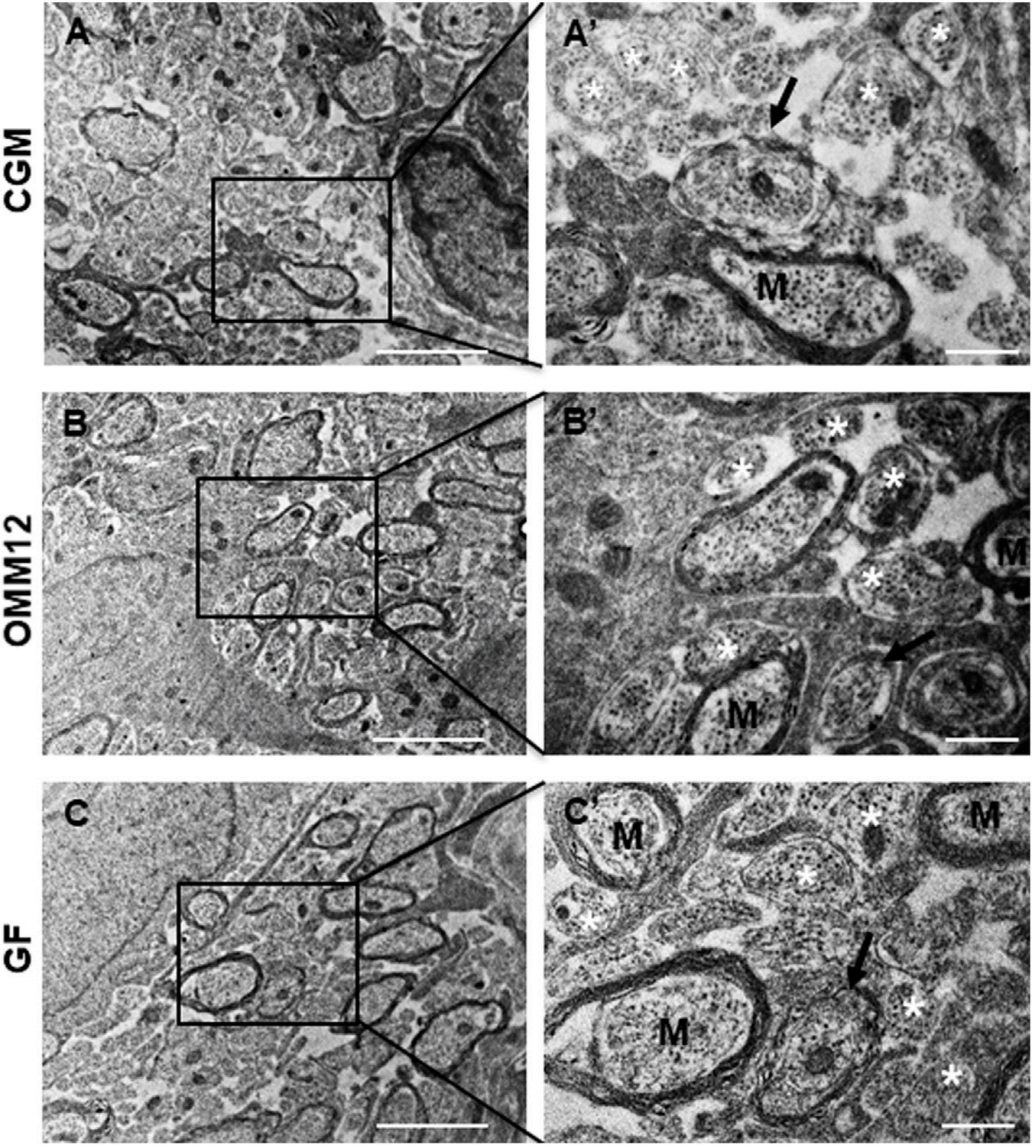
The myelination process is similar among groups. Representative electron microscopy photographs showing the myelination process in the neonatal optic nerves from CGM **(A, A′)**, OMM12 **(B, B′)** and GF **(C, C′)**. At this stage of development (up to 14 days after birth), optic nerves are composed of myelinated fibers (M), unmyelinated axons (white asterisks), and axons enwrapped by a few layers of myelin membrane (black arrows). Scale bar **(A, B, C)** = 2 μm; **(A′, B′, C′)** = 0.5 µm.

The optic nerves of neonatal mice revealed a similar architecture in the three experimental groups (Figures 1A–F), with no visible alterations; stereological and morpho-quantitative analysis performed on myelinated fibers revealed a small decrease in fiber diameter in OMM12 specimens that was significant only in comparison to GF optic nerves (Figure 1K). All the other measured parameters were not statistically different among groups (Figure 1). The morphological results were additionally displayed as regression curves of the distribution of axon diameter (Figure 1N), fiber diameter (Figure 1O), myelin thickness (Figure 1P) and *g*-ratio (Figure 1Q), showing again no major differences among the three groups. Images taken at higher resolution allowed visualizing the state of development and myelination of the optic nerve occurring at about 14 days after birth. A great heterogeneity was observed in all groups, in terms of size and degree of myelination, with properly myelinated axons, along with axons which were still undergoing myelination (myelination appeared still uncompleted) and unmyelinated axons (Figure 2).

**FIGURE 3:**
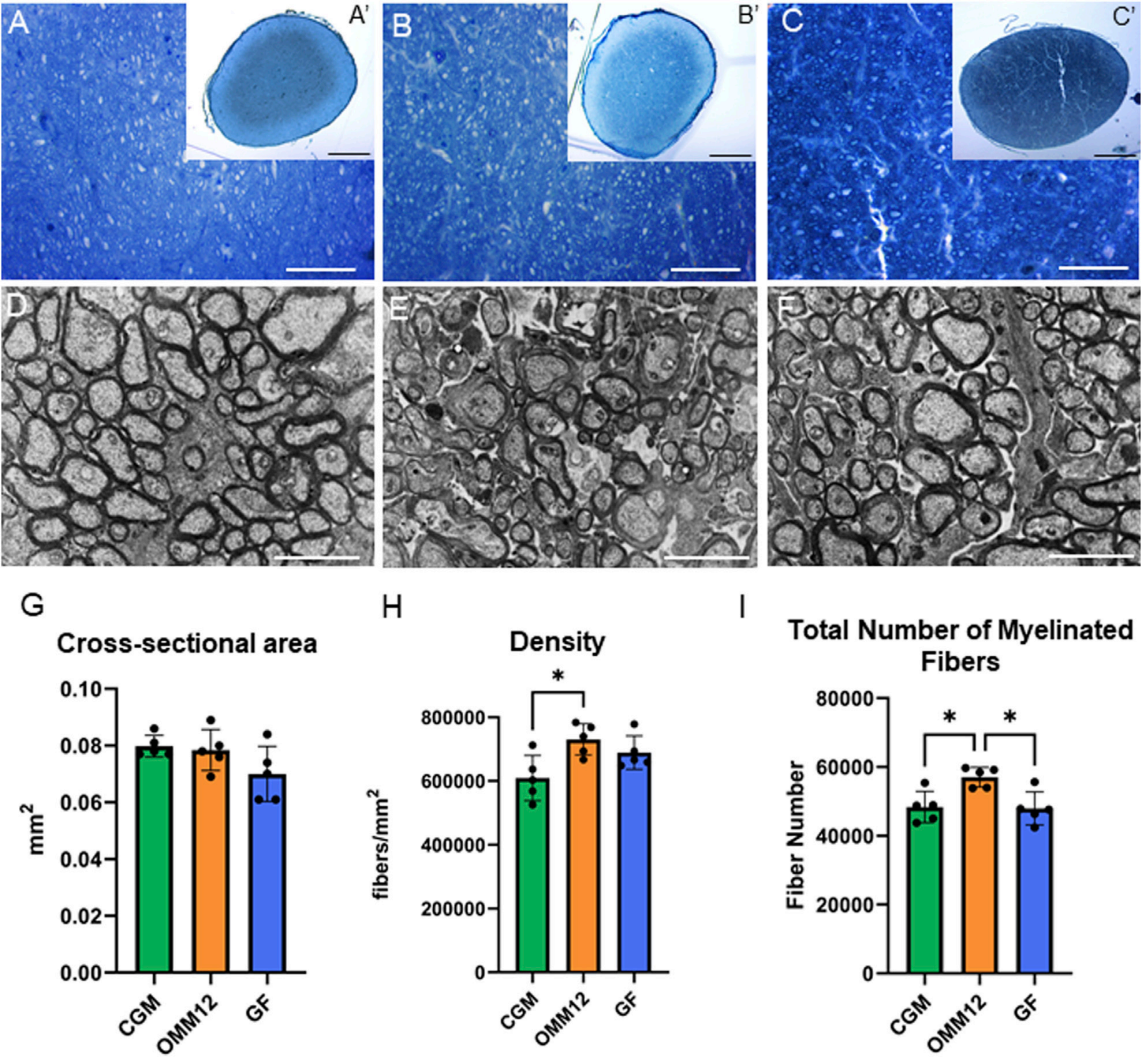
Stereological analysis of young adult optic nerves indicated small differences among groups. **(A–C; A′–C′)** Toluidine blue-stained cross sections of optic nerves obtained from CGM **(A, A′)**, OMM12 **(B, B′)** and GF **(C, C′)** mice, at lower **(A′, B′, C′)** and higher **(A–C)** magnification. **(D–F)** Electron-microscopy images of optic nerves from CGM **(D)**, OMM12 **(E)**, GF **(F)**. **(G–I)** Stereological results revealed significantly higher total number and density of myelinated fibers in OMM12 mice. **(A′, B′, C′)** scale bar = 100 μm; **(A, B, C)** Scale bar = 20 μm; **(D, E, F)** Scale bar = 2 µm. Parametric data were subjected to One-Way ANOVA followed by Tukey’s multiple comparisons *post hoc* test; bar graphs depict the mean ± SEM; *p ≤ 0.05 (n = 5 animals per group).

Light microscopy and low magnification electron microscopy analyses of optic nerves from young adult mice (at about 67 days after birth) revealed, again, a similar organization in the three experimental groups: the optic nerves were composed of closely packed and completely myelinated nerve fibers with variable size (Figures 3A–F). No differences were seen for the cross-sectional area among groups (Figure 3G). Stereological analysis revealed a significantly increased density and higher total number of myelinated fibers in OMM12 optic nerves (Figures 3H, I).

**FIGURE 4:**
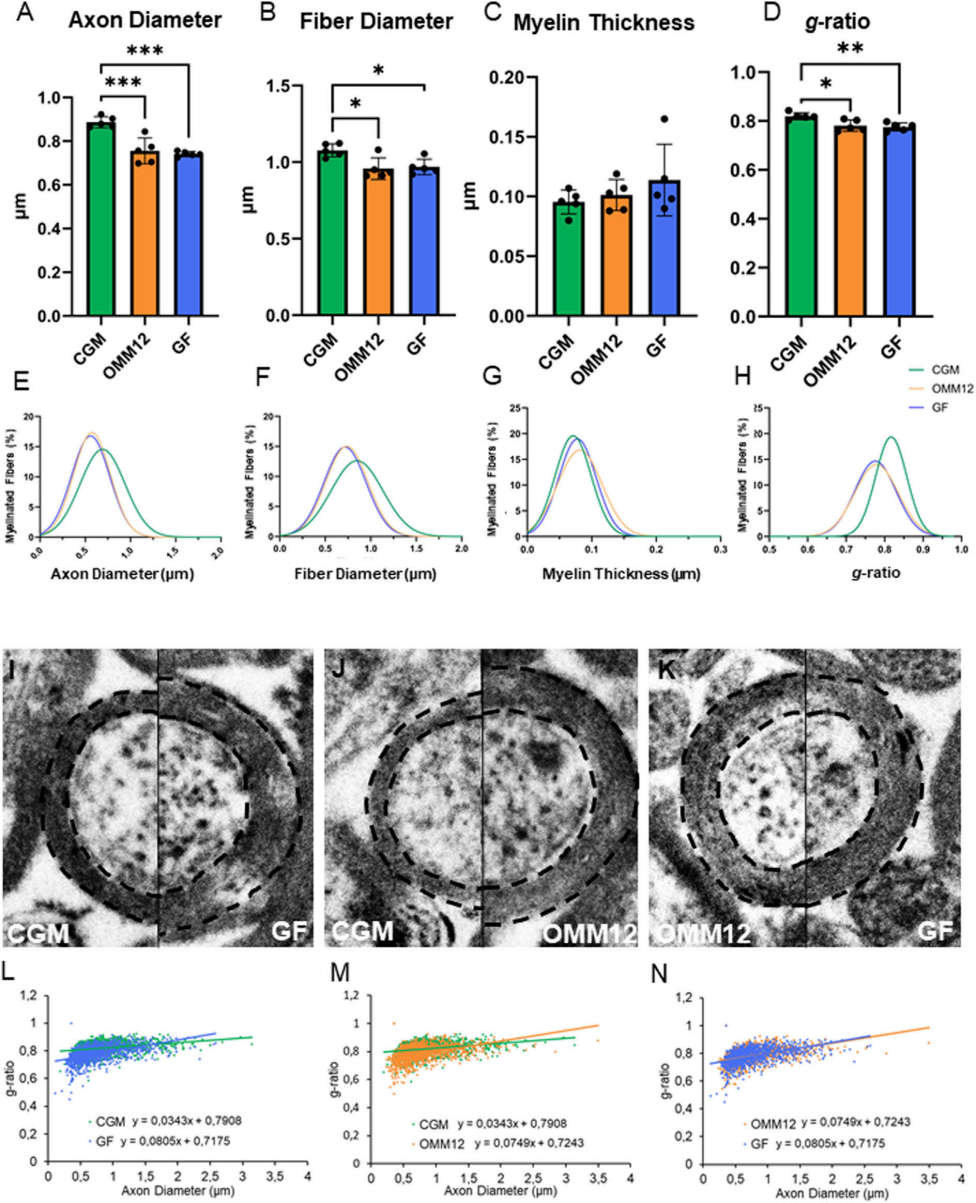
Young adult GF and OMM12 optic nerves displayed smaller and hypermyelinated nerve fibers compared to CGM. **(A–D)** Morpho-quantitative results of young adult optic nerves (n = 5 animals per group). Parametric data were subjected to One-Way ANOVA followed by Tukey’s multiple comparisons *post hoc* test; bar graphs depict the mean ± SEM; *p ≤ 0.05, **p ≤ 0.01, ***p ≤ 0.001. **(E–H)** Regression curves (Gaussian equation, not given) of percentile distribution for axon diameter **(E)**, fiber diameter **(F)**, myelin thickness **(G)** and *g*-ratio **(H)**. **(I–K)** Transmission electron-microscopic comparison of CGM vs. GF **(I)**, CGM vs. OMM12 **(J)** and OMM12 vs. GF **(K)** young adult optic nerve myelinated axons. **(L–N)** Scatter plot graph displaying *g*-ratio (y-axis) in relation to axon diameter (x-axis) of individual fibers (the total number of analysed optic nerve fibers were: CGM n = 1204; OMM12 n = 1450; GF n = 1266).

Morpho-quantitative analysis (Figure 4) revealed significantly reduced axon and nerve fiber dimensions in both young adult GF and OMM12 optic nerves compared to CGM specimens (Figures 4A, B). Interestingly, although the mean thickness of the myelin sheaths, on average, was not significantly but only marginally increased in OMM12 and GF myelinated axons (Figure 4C), the *g*-ratio (defined as the relationship between axon diameter/fiber diameter) was significantly lower in GF and OMM12 samples in comparison to CGM (Figure 4D). Therefore, morpho-quantitative analysis revealed a hypermyelination of myelinated optic nerve fibers in young adult OMM12 and GF mice. Interestingly, the distributions of axon and fiber diameters, displayed as regression curves (Figures 4E, F), showed a clear shift to the left for data derived from GF and OMM12 mice, indicating that optic nerve axons and fibers in these two experimental groups were smaller compared to those in the CGM group. While the regression curve for myelin thickness appears similar across the three experimental groups (Figure 4G), hypermyelination is clearly evident, as indicated by the significant shift to the left in the *g*-ratio data distributions for both OMM12 and GF groups compared to CGM (Figure 4H). This quantitative result was further confirmed by the clearly visible hypermyelinated axons observed in high-magnification electron microscopy images (Figures 4I–K). Finally, the scatter plot of the *g*-ratio against the axon diameter indicated that the decreased *g*-ratio in GF and OMM12 samples compared to CGM was most evident for axons with smaller diameters (Figures 4L–N).

**FIGURE 5:**
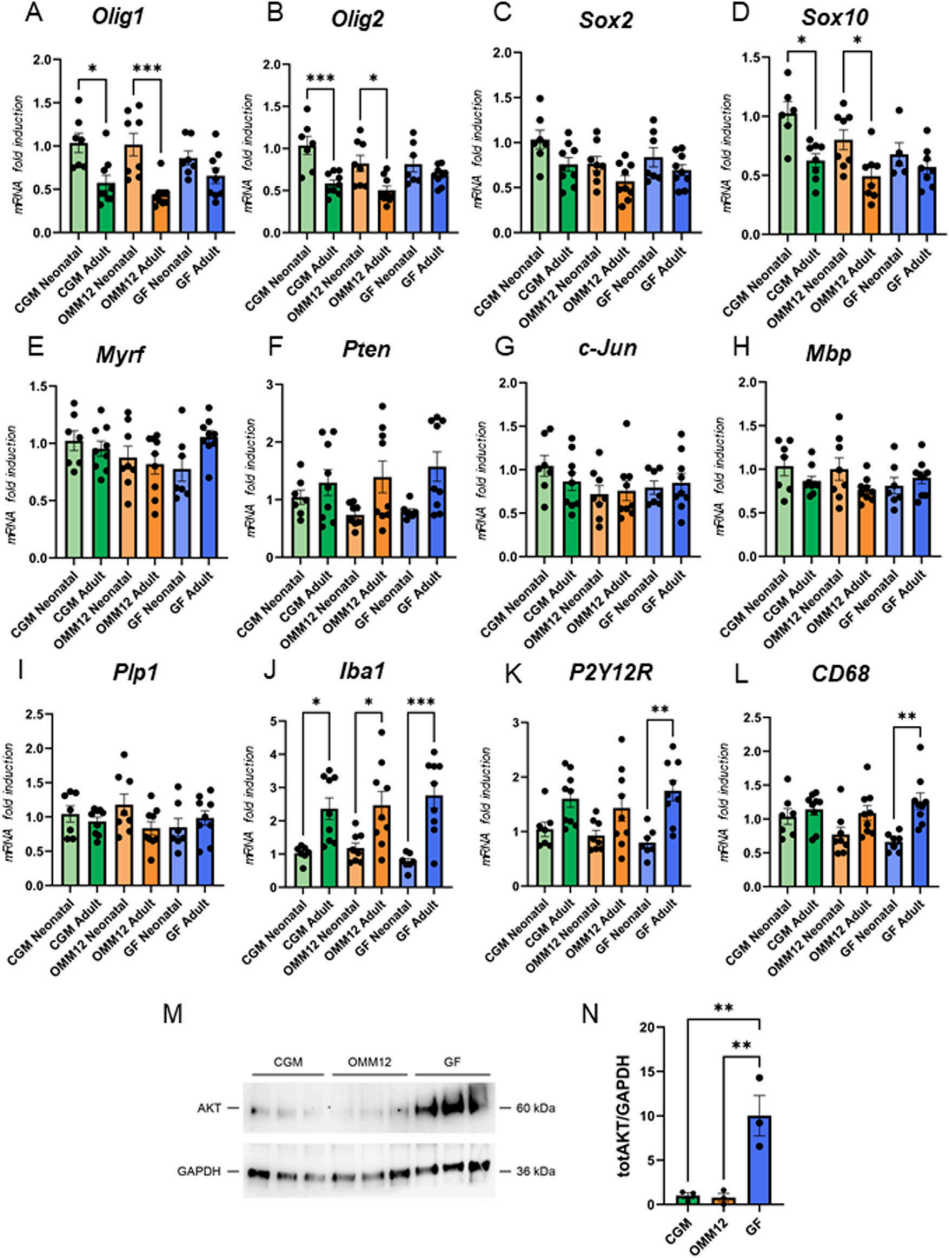
Analysis of gene expression. qRT-PCR analysis for transcripts coding for *Olig-1* **(A)**, *Olig-2,* **(B)**, *Pten* **(C)**, *Myrf* **(D)**, *Sox-2* **(E)**, *Sox-10* **(F)**, *c-Jun* **(G)**, *Mbp* **(H)**, *Plp1* **(I)**, *Iba1* **(J)**, *P2Y12R* **(K)** and *CD68* **(L)** in neonatal and young adult mice optic nerves (neonatal CGM and GF, n = 7; neonatal OMM12, n = 8; young adult CGM, OMM12 and GF, n = 9). **(M)** Western blot analysis of AKT expression levels in protein lysates from young adult optic nerves (n = 3). GAPDH was used as loading control. Protein bands were quantified and AKT expression quantification, normalized to GAPDH, is shown in the graph **(N)**. Normal distribution was tested using the Shapiro-Wilk test. Parametric data were subjected to One-Way ANOVA followed by Tukey’s multiple comparisons *post hoc* test. Bar graphs depict the mean ± SEM; *p ≤ 0.05, **p ≤ 0.01, ***p ≤ 0.001.

To evaluate any differences in gene expression linked to limited or absent gut microbiota, we additionally carried out qRT-PCR on optic nerve samples from both neonatal and young adult mice. Expression levels of genes and transcription factors known to be important for the myelination process are depicted in Figure 5. Our results demonstrated a downregulation of oligodendrocyte transcription factor 1 and 2 (*Olig1* and *Olig2*), as well as sex determining region Y-box 10 (*Sox10*) during maturation of optic nerves in CGM and OMM12 samples, where a lower expression level was evident in young adult optic nerves than in neonatal samples. Interestingly, in the GF specimen only a marginal, but not significant, downregulation was detected (Figures 5A, B, D).

Expression levels of sex determining region Y-box 2 (*Sox2*), myelin regulatory factor (*Myrf*), *c-Jun*, phosphatase and tensin homolog (*Pten*), myelin basic protein (*Mbp*) and proteolipid protein 1 (*Plp1*) showed no differences, neither among the different experimental groups nor in the neonatal versus young adult comparison (Figures 5C, E–I). We also evaluated the expression of microglial markers and we found that ionized calcium-binding adapter molecule 1 (*Iba1*) is upregulated during development in all the three experimental models, while the purinergic P2Y12 receptor (*P2Y12R*) and cluster of differentiation 68 (*CD68*) are significantly upregulated during development only in GF mice (Figures 5J–L).

Since the activation of PI3-kinase and its effector AKT are crucial for initiating myelination in both the PNS (Maurel and Salzer, 2000; Ogata et al., 2004) and the CNS (Flores et al., 2008; Yu et al., 2023), we assessed whether AKT expression and phosphorylation differ among the three experimental groups. Proteins extracted from young adult CGM, OMM12 and GF mice optic nerves were analyzed by Western blotting, as shown in Figure 5M. Total AKT expression normalized to GAPDH expression was significantly higher in GF optic nerves when compared with CGM and OMM12 samples (Figure 5N), while AKT phosphorylation was not detectable (data not shown).

## Discussion

Human gut microbiota is the set of microorganisms that live in the human gut, which evolves throughout life and appears to provide essential health benefits to its host, by producing bioactive metabolites (vitamins, short-chain fatty acids, amino acids and others), regulating energy balance, maintaining immune and metabolic homeostasis and protecting against pathogens (Jandhyala et al., 2015; Rowland et al., 2018). An imbalance in bacterial composition, a condition known as dysbiosis, is becoming recognized as an environmental factor that interacts with the host’s metabolism, causing various disorders (DeGruttola et al., 2016; Hou et al., 2022).

The gut microbiota influences numerous organs and systems via multiple routes including neural, metabolic, endocrine and immune pathways, resulting in the formation of gut-organ axes (Ahlawat and Asha, 2021; Saxami et al., 2023). Within these axes, the gut-brain axis has been recognized since the 19th century (Rutsch et al., 2020). With regard to CNS myelination, most recently a reciprocal interdependence has been described for gut microbiota and oligodendrocytes indicating that the relevant axis could be an interesting therapeutic target (Tang et al., 2024). Furthermore, a growing number of studies have confirmed the existence of the gut–eye axis (Campagnoli et al., 2023) and the gut-retina axis (Zhang and Mo, 2023) emerging with a relevance for pathological conditions of the optic system. Instead, so far research has never been focused on how gut microbiota might modulate the development of the optic nerve in physiological conditions.

To fill this gap, in this study we investigated the impact of the mouse gut microbiota on the rodent optic nerve fiber maturation and myelin development, thus providing additional knowledge on both the gut-eye/retina axis and the gut-brain-axis.

Interestingly, we demonstrated that optic nerves from young adult, but not neonatal, mice completely lacking gut microbiota (germ-free, GF mice) and mice with a limited and strictly controlled microbiota (OMM12 mice) have smaller and significantly hypermyelinated optic nerve fibers, as indicated by an overall decrease in *g-*ratio, compared to mice colonized with a complex gut microbiota (CGM mice). These data are in accordance with studies which revealed that adult GF mice displayed hypermyelinated fibers in the prefrontal cortex, a brain region that shows later myelination than, e.g., primary sensory or motor cortex (Hoban et al., 2016). While this alteration in myelination was originally demonstrated to be unexpectedly region-specific to the prefrontal cortex (Hoban et al., 2016) and also others demonstrated later an impact of gut microbiota on proper callosal myelination (Radulescu et al., 2019), we provide here evidence for further gut microbiota-dependent alterations.

In our analyses we found no alterations in terms of optic nerve fiber myelination in neonatal mice. We can hypothesize that this depends on the observation window and the myelination phase in which we performed the analysis. In the mouse CNS, myelin sheaths are formed by oligodendrocytes, and myelination occurs as a multistep process which includes up to three waves of differentiation of oligodendrocyte progenitor cells into pre-myelinating and mature myelinating oligodendrocytes (Yu et al., 2023). Pre-myelinating oligodendrocytes forming compact myelin sheaths appear at about P7 in the brain, while spinal cord myelination has already initiated at P0. The process of CNS myelination shows a peak between 2 and 4 weeks after birth, and continues for at least eight postnatal months, when myelination is almost completed in most brain regions (Cristobal and Lee, 2022; Yu et al., 2023). A morphometric analysis of the mouse optic nerve conducted in the late 1990s, showed that no myelinated nerve fibers were observed before P5. Once myelination started, it was described to progress very rapidly during the early stages of postnatal development, but to gradually slow down with age, with a peak level of myelination between 9 and 16 weeks of postnatal life (Dangata et al., 1996). A more recent study reports the decisive size of retinal neuron axons for initiating myelination to be >0.3 µm (Mayoral et al., 2018), a size that was already reached by the majority of optic nerve axons in our study (both for neonatal and young adult samples). Furthermore, the authors describe that myelination by pre-myelinating oligodendrocytes continuously increases between P7 and P14 (Mayoral et al., 2018).

With regard to the results we report here, it is likely that we could not detect early postnatal alterations in our P8-14 mice, because we were in this initial and actively changing period of optic nerve myelination with high variation in the number of already myelinated fibers. Furthermore, the optic nerve fibers were very heterogeneous in terms of size and degree of myelination. During stereological and morpho-quantitative analyses, we counted and measured all fibers with a clear myelin sheath, but we were not able to discriminate between the already completely myelinated fibers and the partially myelinated ones. This limitation probably flattened out any variations that could possibly be observed by measuring only the fibers with complete myelin sheaths. We can therefore not definitely exclude that the presence/absence of gut microbiota did not have an effect on the optic nerve myelination already during the first week of optic nerve myelination.

It is noteworthy that we have recently described an effect of the gut microbiota composition on the myelination in the somatic PNS, where nerve fibers are myelinated by another type of myelinating glial cells, namely, the Schwann cells (Cescon et al., 2024). Axons in the peripheral median nerves of GF and OMM12 mice were also detected to be reduced in axonal diameter while myelin thickness was increased, resulting in hypermyelinated axons. This indicates that central and peripheral axons as well as central and peripheral glia may share at least parts of their myelination-inducing cross-talk (Harty et al., 2019), resulting in a similar gut microbiota-dependent effect on myelination. The underlying mechanisms leading to the described similar phenotype in optic and somatic peripheral nerves remain to be elucidated. Additionally, no phenotypic rescue is observed in either the median or optic nerve in OMM12 mice, suggesting that the 12 bacterial species colonizing the gut in this specific gnotobiotic model are insufficient to support optimal maturation and proper myelination in either the somatic PNS or the optic nerve.

Myelination is a process tightly regulated during development by both cellular and molecular factors, such as transcription factors, growth factors, chemokines/cytokines, axonal signals and intracellular signaling pathways (Yu et al., 2023; Sock and Wegner, 2019; Simons et al., 2024). To understand whether some of these key factors were altered and therefore could explain the hypermyelinated phenotype of retinal ganglion cell axons in the optic nerves of GF and OMM12 mice, we analyzed the expression levels of different genes involved in the regulation of oligodendrocyte development and myelination in both neonatal and young adult samples.

Our results showed a downregulation of the transcription factors *Olig-1/2* and *Sox10* between neonatal samples and their young adult counterparts from CGM and OMM12 mice; however, this downregulation was not observed in GF animals, where similar expression levels were detected between neonatal and young adult optic nerves. Therefore, we have to assume that while OMM12 and GF mice exhibited phenotypic similarity in terms of ultrastructural hypermyelination, they differed in gene expression (at least for the genes we analyzed in our study). This leads us to hypothesize that colonization with the 12 bacterial species in the OMM12 mouse model was sufficient to rescue transcriptomic changes, but not phenotypic changes, in the optic nerve. In accordance with this is the report that colonization of GF mice with conventional gut microbiota at weaning was sufficient to rescue transcriptomic changes in the prefrontal cortex, but it could not reverse the hypermyelinated axonal phenotype (Hoban et al., 2016). The authors suggested a critical time window in which gut microbiota signaling could be necessary for regular cortical myelination (Hoban et al., 2016). From our results, we hypothesize that, in the very first weeks of embryonic development and very first weeks of postnatal life, the expression of *Olig1*, *Olig2* and *Sox10*, as positive regulators of oligodendrocyte maturation, differentiation and myelination (Sock and Wegner, 2019) is increased, to allow a correct myelination of nerve fibers. Subsequently, in a later phase, when myelination is completed, in the animals with physiological (CGM) or limited (OMM12) gut microbiota these transcriptional factors become downregulated, but not in the GF animals; therefore, the absence of microbiota seems to lead to a stimulation of myelination for a more prolonged period, through mechanisms yet to be investigated.

Moreover, the analysis of markers of microglia, the resident immune cells involved in regulatory processes critical for development and physiology of the CNS (Cook and Prinz, 2022), revealed an increase in *Iba1* expression during optic nerve development across all three experimental models, while *P2Y12R* and *CD68* are significantly upregulated during development only in GF mice. This transcriptomic difference suggests a potential functional variation in microglial activity in mice lacking gut microbiota, as previously demonstrated in brain tissue (Luck et al., 2020), which could impact the myelination process. Indeed, the distinct properties of microglia enable them to regulate myelination both during development and throughout life (Santos and Fields, 2021).

Derived from what has been described in the literature, it is possible that events of hypermyelination in the maturing optic nerve depend on a disruption of dynamic neuronal signaling along the fibers that undergo myelination (Mayoral et al., 2018). In addition, evidence exists that gut microbial metabolites within the circulating metabolome develop an impact on neuronal signaling and modulate brain activity (Ahmed et al., 2022). Therefore, and with appropriate caution, we can carefully speculate that missing metabolites from the gut microbiota account for a dysregulation in optic nerve myelination in our study. We consider that the OMM12 gut microbiome still lacks important components that could sufficiently restore the axon-oligodendrocyte cross-talk towards correct myelination of optic nerve fibers. The search for critical bacterial strains or metabolites and the direct link between the same and optic nerve myelination must be the focus of future studies.

In conclusion, our results demonstrate a critical role for the gut microbiota in driving optic nerve maturation and myelination, and therefore contribute to extending our knowledge in the gut-eye/gut-retina-axis.

## Data Availability

The raw data supporting the conclusions of this article will be made available by the authors, without undue reservation.
